# A Suggestion for the Laboratory

**Published:** 1882-09

**Authors:** 


### A SUGGESTION FOR THE LABORATORY.

As the dentist's laboratory is usually located in the dwelling, it is advisable that all noise may be prevented as much as possible.

It has been suggested that rubber cushions be placed under the legs of the work-bench, and in a certain factory it is stated that by this device the hammering of fifty coppersmiths was scarcely audible in the room below. Kegs of sand or sawdust are applied in the same way. A few inches of sand or sawdust is at first pounded into each keg; on this is laid a board or block upon which the leg rests, and around the leg and block is pounded fine dry sand or sawdust. Not only all noise, but all vibration and shock are prevented; and an ordinary anvil so mounted may be used in a dwelling house without the least annoyance to the inmates. To amateur workmen and dentists whose workshops are located in dwelling-houses, this device affords a cheap and simple relief from the annoyance of noise.—*Dental Office and Laboratory.*

